# On-farm performance and farmers’ participatory assessment of new stress-tolerant maize hybrids in Eastern Africa

**DOI:** 10.1016/j.fcr.2019.107693

**Published:** 2020-02-01

**Authors:** Mosisa Worku, Hugo De Groote, Bernard Munyua, Dan Makumbi, Fidelis Owino, Jose Crossa, Yoseph Beyene, Stephen Mugo, McDonald Jumbo, Godfrey Asea, Charles Mutinda, Daniel Bomet Kwemoi, Vincent Woyengo, Michael Olsen, Boddupalli M. Prasanna

**Affiliations:** a International Maize and Wheat Improvement Center (CIMMYT), P. O. Box 1041, Village Market, 00621, Nairobi, Kenya; b CIMMYT, Apdo. Postal 041, C.A.P. Plaza Galerías, Col. Verónica Anzures, 11305 Ciudad de México, Mexico; c National Crops Resources Research Institute (NaCRRI), P.O. Box 7084, Namulonge, Uganda; d Embu Research Center, Kenya Agricultural and Livestock Research Organization (KALRO), P.O. Box 27, 60100, Embu, Kenya; e Kakamega Research Center, Kenya Agricultural and Livestock Research Organization (KALRO), Kakamega, P.O. Box 169, 50100, Kenya

**Keywords:** Grain yield, Participatory evaluation, Stability, Stress tolerance, Trait preference

## Abstract

The development and deployment of high-yielding stress tolerant maize hybrids are important components of the efforts to increase maize productivity in eastern Africa. This study was conducted to: i) evaluate selected, stress-tolerant maize hybrids under farmers’ conditions; ii) identify farmers’ selection criteria in selecting maize hybrids; and iii) have farmers evaluate the new varieties according to those criteria. Two sets of trials, one with 12 early-to-intermediate maturing and the other with 13 intermediate-to-late maturing hybrids, improved for tolerance to multiple stresses common in farmers’ fields in eastern Africa (drought, northern corn leaf blight, gray leaf spot, common rust, maize streak virus), were evaluated on-farm under smallholder farmers’ conditions in a total of 42 and 40 environments (site-year-management combinations), respectively, across Kenya, Uganda, Tanzania and Rwanda in 2016 and 2017. Farmer-participatory variety evaluation was conducted at 27 sites in Kenya and Rwanda, with a total of 2025 participating farmers. Differential performance of the hybrids was observed under low-yielding (<3 t ha^−1^) and high-yielding (>3 t ha^−1^) environments. The new stress-tolerant maize hybrids had a much better grain-yield performance than the best commercial checks under smallholder farmer growing environments but had a comparable grain-yield performance under optimal conditions. These hybrids also showed better grain-yield stability across the testing environments, providing an evidence for the success of the maize-breeding approach. In addition, the new stress- tolerant varieties outperformed the internal genetic checks, indicating genetic gain under farmers’ conditions. Farmers gave high importance to grain yield in both farmer-stated preferences (through scores) and farmer-revealed preferences of criteria (revealed by regressing the overall scores on the scores for the individual criteria). The top-yielding hybrids in both maturity groups also received the farmers’ highest overall scores. Farmers ranked yield, early maturity, cob size and number of cobs as the most important traits for variety preference. The criteria for the different hybrids did not differ between men and women farmers. Farmers gave priority to many different traits in addition to grain yield, but this may not be applicable across all maize-growing regions. Farmer-stated importance of the different criteria, however, were quite different from farmer- revealed importance. Further, there were significant differences between men and women in the revealed-importance of the criteria. We conclude that incorporating farmers’ selection criteria in the stage-gate advancement process of new hybrids by the breeders is useful under the changing maize-growing environments in sub-Saharan Africa, and recommended to increase the turnover of new maize hybrids.

## Highlights

• New stress-tolerant maize hybrids were tested in four East African countries.
• They were evaluated by 2,0252025 farmers (55% women) at 27 sites in Kenya and Rwanda.
• New hybrids had 18% higher yields than commercial checks under farmers’ conditions.
• Their yields were similar to those of commercial checks under optimal conditions.
• Farmers generally gave the highest scores to the hybrids with the highest yields.

## 1 Introduction

Maize is one of the major staple crops in East Africa. on which millions of smallholder farmers depend their food security and livelihood (Beshir et al., 2012a, 2012b; Cairns and Prasanna, 2018). East Africa accounts for 27% of the maize area and 28.3% of the maize grain production in Africa (FAOSTAT, 2017). In Africa, maize yields only increased from 1 t ha^−1^ in 1961 to 1.9 t ha^−1^ in 2016, in eastern Africa only increased from about 1 t ha^−1^ to 2.1 t ha^−1^ (an increase of 2% per year), very low compared to the world average of 5.6 t ha^−1^ (FAO, 2018).

Adoption of improved maize varieties in the study region vary widely among countries, from 34% in Tanzania, and 54% in Uganda, to 69% in Kenya (De Groote et al., 2015). Despite high levels of adoption of improved maize seed in Kenya (Hassan et al., 1998), national maize yield in Kenya remains quite low compared to other eastern African countries. Major reasons for this stagnation and low maize yield abiotic stresses, such as poor soil-fertility and frequent drought (Binswanger-Mkhize and Savastano, 2017), and biotic stresses such as maize lethal necrosis disease (MLN), northern corn leaf blight, gray leaf spot, common rust, maize streak virus (MSV), ear rot, *Striga*.), stem borers, and more recently fall armyworm (Cairns and Prasanna, 2018; Keno et al., 2018; Prasanna et al., 2018). Coupled with these are socioeconomic factors, including inaccessibility of farm inputs and credit, poor agronomic management (especially low fertilizer application, suboptimal or untimely weedings, late planting, and low plant population), and poor market access for smallholder farmers (Beshir, 2012; Cairns and Prasanna, 2018).

Recognizing the need for improving maize yields in eastern and southern Africa, not only through enhanced yields but also through improved resilience to various abiotic and biotic stresses, the International Maize and Wheat Improvement Center (CIMMYT), in collaboration with public and private partners, has embarked on intensive maize-breeding programs in the region (Banziger and Diallo, 2004; Banziger et al., 2006; Beyene et al., 2017). The key strategy is the use of managed abiotic and biotic stress screening sites, in which selections are carried out under managed stress as well as under random stress and optimal conditions (Bänziger et al., 1999; Worku et al., 2012; Makumbi et al., 2018). Over the last three decades, several projects implemented by CIMMYT and its partners have resulted in a large number of high-yielding and multiple-stress-tolerant maize varieties now commercialized in eastern sub_Saharan Africa (SSA) (Beyene et al., 2013; Setimela et al., 2017; Makumbi et al., 2018). Still, varietal turnover in SSA remains low; the weighted average age of hybrid maize varieties grown in SSA is 13 years, much higher than the average in other parts of the world, 5–8 years (Abate et al., 2017).

Therefore, it is crucial to study genetic gains under farmers’ conditions to monitor the efficiency of varietal turnover and to refine the product development and deployment strategies. Genetic gain studies of CIMMYT’s elite maize hybrids in eastern and southern Africa (ESA), developed from 2000 to 2010 under different on-station managed stressed and optimal conditions, showed that the highest gain for grain yield of the new varieties: 141.3 kg ha^−1^ yr^−1^, was found for the hybrids subjected to the stress of maize streak virus, compared to a gain of 109.4 kg ha^−1^ yr^−1^ under optimal conditions (Masuka et al., 2017). Genetic gains for other stresses were 32.5 kg ha^−1^ yr^−1^ under managed drought stress, 20.9 kg ha^−1^ yr^−1^ under low N, and 22.7 kg ha^−1^ yr^−1^ under random stress, indicating the need for intensive efforts to accelerate and improve genetic gains. Setimela et al. (2017) evaluated 20 elite maize hybrids under on-farm conditions in ESA from 2011 to 2012 and reported a 4–19% grain yield advantage of the new stress-tolerant (ST) maize hybrids over the popular commercial checks. The superior performance of these new maize hybrids evaluated under researcher-managed on-station conditions needs to be verified under on-farm smallholder farmers’ conditions, as farmers are the ultimate users of the new maize hybrids.

Abate et al. (2017) observed that despite the release of new maize varieties in SSA between 2000 and 2014, the average age of the oldest released variety in those 24 countries was 20 years while the average age of the youngest was seven years. While there could be many reasons for the slow varietal turnover in SSA, researchers need to engage intensively with farmers, study their needs under the changing environments, incorporate farmer-desired traits in the breeding pipeline, and improve varietal turnover through intensive engagement with the seed companies. This will assist in the wider adoption of new maize hybrids (Morris and Bellon, 2004; Setimela et al., 2018). One approach is farmer participatory variety selection, which has been applied for some time in the maize breeding programs in eastern Africa

Conceptually, farmers are the end-users of maize research products, especially the improved varieties. Farmers, like consumers, derive satisfaction not from the goods themselves, but from the attributes these goods provide (Lancaster, 1966). Participatory evaluation, therefore, consists of two components: i) identifying the attributes or traits important to famers and the extent of their importance to farmers, and ii) evaluating the varieties with regard to the identified traits as well as to the overall products.

Experience has shown that breeding programs can benefit from participatory approaches, starting with a good understanding of farmers' knowledge about their crop varieties (Haugerud and Collinson, 1990). Formally, these approaches can be categorized into Participatory Varietal Selection (PVS), the identification of farmer-preferred cultivars, and Participatory Plant Breeding (PPB) (Witcombe et al., 1996), where PPB is a logical extension of PVS (Joshi and Witcombe, 1998). Several studies have shown that PVS can increase the adoption of improved varieties (Sperling et al., 1993; Joshi and Witcombe, 1996; Defoer et al., 1998) and hence increase productivity (Witcombe, 1999) and that PPB is more likely to produce farmer-acceptable products, particularly for marginal environments (Witcombe et al., 1996; Atlin and Cooper, 2001).

Many studies have shown the potential of participatory approaches. In India, a review of PVS in different crops showed that farmers assess varieties on a wider set of farmer-relevant parameters than those measured in plant breeders' trials, and that farmers rarely prefer the released, recommended cultivars (Joshi and Witcombe, 1998). Still in India, PVS on rice and chickpea showed that while some farmer-acceptable varieties were found amongst released varieties, none were among those recommended for the area (Joshi and Witcombe, 1998); in another programme one variety highly preferred by farmers spread quickly and widely in the target region (Witcombe et al., 2001). In Nepal, a PPB programme for rice showed how the varieties most preferred by farmers were adopted rapidly, and that the most adopted variety performed much better than those from conventional breeding (Sthapit et al., 2008).

Participatory research on maize also has a long history. In southern Africa, the “mother and baby” trial design was developed in collaborative plant breeding approaches (Snapp, 2002), and the results showed the latter have an advantage over traditional approaches in selecting and deploying more appropriate varieties (Bänziger and de Meyer, 2002). In East Africa, participatory research on maize varieties started in 1999 in Kenya (Siambi et al., 2002), and a methodology was developed based on identifying criteria relevant to farmers, and scoring the varieties being tested on those criteria (De Groote et al., 2002), which is used till today. In Asia, plant breeders and farmers worked together to produce improved maize varieties for the low-resource farmers of Gujarat, India, and the returns from PPB were higher than conventional breeding because it is cheaper and benefits to farmers are realized earlier (Witcombe et al., 2003). In Nepal, acquisition of local knowledge and participation of farmers in PVS and PPB enabled rapid identification and development of adapted maize germplasm with significantly higher yield than extant varieties (Tiwari et al., 2009).

It is also important to understand whether the farmers’ preferences differ among target agroecological zones (AEZs), such as high potential vs low potential areas, and among different socioeconomic groups, in particular by gender, age, education and wealth. Participatory rural appraisals or focus group discussions are useful to ask farmers to list, rank and weigh up the different traits they use to evaluate and select varieties (Almekinders and Elings, 2001). In regions where such exercises have already been conducted, this list of traits can be added to the main questionnaire, and farmers can be asked to score the traits for their importance (Coe, 2002).

Based on these principles and on recent experience, the present study was designed with the following objectives: i) to evaluate selected stress-tolerant maize hybrids developed by CIMMYT in eastern Africa under farmers’ conditions; ii) to identify farmers’ selection criteria in evaluating and selecting maize hybrids; iii) to let farmers evaluate the varieties and score them for the identified criteria and overall. In a novel approach, we also compared the importance of the different criteria, as stated by farmers, with the importance as revealed by regressing the overall evaluation score on the scores for the individual criteria, interpreting the coefficients as a weight or level of importance.

## 2 Materials and methods

### 2.1 Overview

On-farm trials with new ST maize hybrids were organized in Kenya, Uganda, Tanzania and Rwanda in 2016 and 2017. Varieties in all trials were evaluated for yield and other biophysical characteristics and compared to commercial and internal checks, while farmer evaluations were conducted in a subset of trials from Kenya and Rwanda.

### 2.2 Experimental hybrids used in the trials

For this study, two sets of varieties, one with early-to-intermediate maturing hybrids and the other with intermediate-to-late maturing hybrids, were evaluated on-farm under smallholder farmers’ conditions All the entries in both sets of trials, with the exception of the control, were improved for tolerance to multiple stresses common on farmers’ field in East Africa (drought, northern corn leaf blight, gray leaf spot, common rust, maize streak virus). The first set included 12 early-to-intermediate maturing hybrids, while the second set had 13 intermediate-to-late maturing hybrids, including popular commercial checks selected by the farmers (Table 1). The early-to-intermediate set comprised seven pre-commercial CIMMYT test hybrids, two internal genetic gain checks, two popular commercial hybrid checks (selected by CIMMYT), and one farmer’s check variety (selected by the farmer in each site). The intermediate-to-late set included six pre-commercial CIMMYT test hybrids, three internal genetic gain checks, three popular commercial checks, and one farmer’s check variety. The internal genetic gain checks were the best recent hybrids developed by CIMMYT and were either commercially grown by farmers or in the process of commercialization. The farmer’s check differed among the sites for the two trial sets.

**Table 1:** Early-to-intermediate and intermediate-to-late maturity maize hybrids used in the on-farm trials in East Africa, 2016–2017.

	Early-to-intermediate varieties		Intermediate-to-late varieties	
Entry^a^	Hybrid	Source	Hybrid	Source
1	CKH122114	CIMMYT	CKH13605	CIMMYT
2	EMH1101	CIMMYT	CKH143770	CIMMYT
3	CKH143975	CIMMYT	WE2106	CIMMYT
4	WE3102	CIMMYT	WE3104	CIMMYT
5	WE4120	CIMMYT	KM1201	CIMMYT
6	WE3106	CIMMYT	WE3105	CIMMYT
7	WE4109	CIMMYT	CKH10769	CIMMYT (internal check)
8	WE3101	CIMMYT (internal check)	CZH0837	CIMMYT (internal check)
*9*	DLSH103	CIMMYT (internal check)	WE1101	CIMMYT (internal check)
*10*	DUMA43	Commercial check^b^	PHB30G19	Commercial check
*11*	PAN4M-19	Commercial check	WH505	Commercial check
12	Farmers' check **	Farmers^c^	WH509	Commercial check
13			Farmers' check *	Farmers

a Average days to anthesis for the entries was 56.5 days (779.6 °C growing degree days) and 59.5 days (813.4 °C growing degree days) at Kiboko, 960 m above sea level, for early-to-intermediate and intermediate-to-late maturity groups.

b Each farmer used mainly commercial hybrids popular in his area as check in both trials.

c Farmer check (Tanzanian farmers selected SC627; Kenya farmers selected WH403, H513, Embu Synthetic, H517, H520, KH-500-13-E, or KH600-14-E; Ugandan farmers selected Longe 7H, Longe 11H, while Rwandan farmers selected SC513, SC403).

All the new hybrids were three-way crosses developed by CIMMYT maize-breeding programs in Kenya and Zimbabwe (as were the internal checks), andselected based on performance in on-station trials conducted across locations in eastern Africa under managed drought, random drought, low N, and optimal conditions, as described elsewhere (Odiyo et al., 2014; Beyene et al., 2016; Makumbi et al., 2018).

### 2.3 Evaluation of yield and biophysical performance of the new hybrids

#### 2.3.1 Experimental sites, experimental design, and trial management

**Fig. 1:**
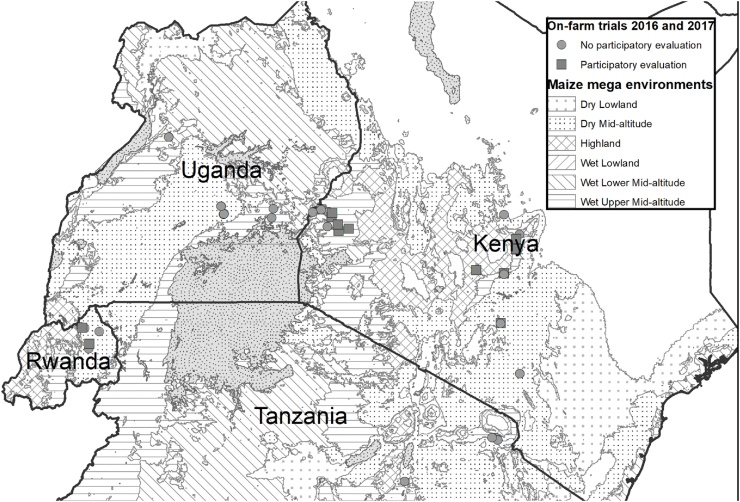
Map showing the distribution of regional on-farm trials sites, 2016–2017, superimposed on maize mega environments (sources: authors, CIMMYT, Sonder, 2016).

The on-farm trials were conducted in 2016 and 2017 in Kenya, Uganda, Tanzania, and Rwanda under rainfed conditions and farmers’ management, except at the KALRO-Kiboko demonstration site where supplementary irrigation was used (Fig. 1). All the trials were planted in both years in the target agro-ecologies (where the elevation ranges from 960 to 1776 m above sea level - masl). The hybrids in the early-to-intermediate maturity set were evaluated at a total of 60 environments (site-year-management combinations), while those in the intermediate-to-late maturity set were evaluated at a total of 54 environments. However, grain-yield data were collected successfully at only 42 environments for the early-to-intermediate set and 40 environments for the intermediate-to-late set (Table 2), while the other sites were discarded because of either attack by fall armyworm, particularly in Uganda, damage due to wild animals, or poor stand caused by inadequate soil moisture after planting. A randomized complete block design with sites as replicates was used (Schmidt et al., 2018). Each entry was planted as per recommendation in six rows of 5 m length, with a spacing of 0.75 m between rows and 0.25 m between plants, or approximately 53,333 plants ha^−1^ (Makumbi et al., 2018). Trials were hand-planted with two seeds per hill and thinned to one plant per hill two weeks after planting.

**Table 2:** Number of sites where bio-physical and participatory evaluations were conducted in East Africa, 2016–2017 (C = Central, E = Eastern, W = Western, MA = mid-altitudes, T = transitional, E–I = early-to-intermediate, I–M = intermediate-to-late maturity.

			Kenya					Rwanda	Tanzania	Uganda		Total	
		Characteristic	C-MA	E-MA	ET	W-MA	All	MA					
*Bio-Physical data sites*													
	E-I group	N sites, 2016	2	3	1	5	11	2	2		4		19
		N sites, 2017	2	3	3	6	14	2	2		5		23
		N sites, total	4	6	4	11	25	4	4		9		42
	I-L group	N sites, 2016	2	3	1	5	11	2	2		4		19
		N sites, 2017	2	3	1	6	12	2	2		5		21
		N sites, total	4	6	2	11	23	4	4		9		40
*Participatory variety evaluation sites*													
	E-I group	N sites, 2016	1	2	0	2	5	0					5
		N sites, 2017	2	2	1	4	9	1					10
		N sites, total	4	3	1	4	12	1					13
	I-L group	N sites, 2016	1	1	0	2	4	0					4
		N sites, 2017	2	2	0	4	8	2					10
		N sites, total	3	3	0	6	12	2					14
	Participants	N 2016	110	118	0	234	462	0					462
		N 2017	294	227	123	741	1385	178					1563
		N total	404	345	123	975	1847	178					2025
		Women (%) 2016	56	63		42	51	0					51
		Women (%) 2017	69	63	41	51	56	45					55
		Women (%) total	66	63	41	49	55	45					54
*Yields (tonnes/ha)*													
	PVE sites	Mean	6.52	6.79	2.22	5.02	5.72	5.72					5.77
		(st. dev.)	(1.83)	(1.87)	(0.95)	(1.74)	(2.07)	(2.07)					(2.17)
	Other sites	Mean		4.14	1.12	5.14	5.19	4.52	2.40		2.52		3.61
		(st. dev.)		(2.76)	(0.68)	(1.93)	(2.74)	(2.45)	(2.49)		(1.74)		(2.57)
	All sites	Mean	6.52	5.48	1.67	5.08	5.47	5.21	2.40		2.68		4.39
		(st. dev.)	(1.83)	(2.7)	(0.98)	(1.83)	(2.43)	(2.32)	(2.49)		(1.74)		(2.64)

All trials were managed by the farmers who followed their own practices. All trials except for Rwanda were planted between March 21 and May 3 and harvested between August 14 and October 31, in both years. The trials in Rwanda which were planted between September 27 and October 3, and harvested between March 20 and April 15 in 2017, and between February 13 and March 5 in 2018. Weeds were controlled by hand. Fall armyworm was controlled by the application of a chemical insecticide (ESCORT® 19EC, Jingsu United Agrochemicals Co. Ltd, China) at the rate of 400−500 ml ha^−1^ in 1000 l water at the three sites around Kakamega in western Kenya where it was observed in 2017. Fertilizer application rates (by approximation: 60 kg P2O5 ha^−1^ and 63 kg N ha^−1^ in Kenya, 60 kg P2O5 ha^−1^ and 58 kg N ha^−1^ in Uganda, 40 kg P2O5 ha^−1^ and 60 kg N ha^−1^ in Tanzania, 75 kg P2O5 ha^−1^ and 92 kg N ha^−1^ in Rwanda), and other cultural practices were carried out by farmers according to their practices at each site.

#### 2.3.2 Grain yield data collection

For each entry, field weight was recorded from all ears in the four central rows (15 m2). A sample of ears was shelled, and grain moisture was recorded using DICKEY-john multi-grain moisture tester (Dickey-John Corporation, USA). Grain yield (t ha^−1^) was estimated from field weight using an average shelling percentage of 80% and adjusted to 12.5% moisture.

To compare performance of the hybrids in high and low potential environments, the environments were separated by their average grain yields, with a cut-off of 3 t ha^−1^. The environments with a mean grain yield <3 t ha^−1^ were considered low-yielding environments (13 for the early-to-intermediate maturity set and 9 for the intermediate-to-late maturity set), those with a mean grain yield >3 t ha^−1^ as high yielding or optimal environments (29 for the early-to-intermediate set and 31 for the intermediate-to-late maturity set) (Weber et al., 2012).

#### 2.3.3 Statistical analyses of grain-yield data

Statistical analyses employed a linear fixed effect model for both maturity categories under the two management levels (>3 t ha^−1^) and (<3 t ha^−1^). The linear model for each management level had a general mean and the fixed effects of the hybrids and environments (site-year combinations). The linear fixed effect model for the combined analyses across the two management levels included the fixed effects of the hybrids, management level, and environments. To understand the combined effect of hybrids, management and hybrids × management, and for clustering management levels, we used the sites regression model (SREG) (Crossa and Cornelius, 1997). The SREG model is briefly described as follows.

The basic conventional fixed effect linear model for describing the mean response of hybrids in management, and for studying and interpreting hybrid × management is

$${\bar{y}}_{ij}=\text{μ}+{\text{τ}}_{i}+{\text{δ}}_{j}+{\left(\text{τ δ}\right)}_{ij}+{\bar{\varepsilon }}_{ij}$$

where ${\bar{y}}_{ij}$ is the empirical mean response of the ith hybrid (i = 1,2,…,I) in the jth management (j = 1,2,…,J) with *r* replications in each of the I × J cells, μ is the grand mean over all ${\text{τ}}_{\text{i}}$ is the effect of the ith hybrid, ${\text{δ}}_{\text{j}}$ is the effect of the jth management, ${\left(\text{τ δ}\right)}_{\text{ij}}$ is the interaction of the ith hybrid in the jth management and ${\bar{\varepsilon }}_{ij}$ is the (average) error.

When the effect of the i^th^ hybrid and the effect of the interaction of the i^th^ hybrid in the j^th^ management ${\left(\text{τ δ}\right)}_{ij}$ are estimated together and subjected to singular value decomposition, the above linear model can be expressed as

$${\bar{\text{y}}}_{\text{ij}}=\text{μ}+\delta_{\text{j}}+\sum_{\text{m}=1}^{\text{t}}{\text{λ}}_{\text{m}}{\text{α}}_{\text{im}}{\text{γ}}_{\text{jm}}+{\bar{\text{ε}}}_{\text{ij}}\;\;$$

where the constant ${\text{λ}}_{\text{m}}$ is the singular value of the m^th^ multiplicative component, which is ordered ${\text{λ}}_{1}\geq {\text{λ}}_{2}\geq ...\geq {\text{λ}}_{\text{t}}$; ${\text{α}}_{\text{im}}$ corresponds to the left singular vector of the m^th^ component and represents the hybrid sensitivities to hypothetical management factors represented by the right singular vector of the m^th^ component, ${\text{γ}}_{\text{jm}}$. The ${\text{α}}_{\text{im}}$ and ${\text{γ}}_{\text{jm}}$ satisfy the ortho-normalization constraints $\sum_{\text{i}}{\text{α}}^{\text{im}}{\text{α}}^{\text{im'}}=\sum_{\text{j}}{\text{γ}}^{\text{jm}}{\text{γ}}^{\text{jm'}}=0$ for m ≠ m′ and $\sum_{\text{i}}{\text{α}}_{\text{im}}^{2}=$ $\sum_{\text{j}}{\text{γ}}_{\text{jm}}^{2}=1\text{.}$ This model is named the Site Regression model (Crossa and Cornelius, 1997).

The results of the Site Regression models can be presented graphically in the form of biplots (Gabriel, 1978), in which the hybrid and managements level scores of the first two bilinear terms are represented by vectors in a space, with starting points at the origin and end points determined by the scores. The hybrids and management scores of the first and second bilinear terms are plotted. The distance between two hybrid vectors (their end points) is indicative of the amount of interaction between the hybrids.

### 2.4 Participatory farmers’ evaluation and socio-economic data collection

#### 2.4.1 Principles

To evaluate new maize hybrid varieties, farmers are invited to observe them in the field, typically twice: at mid-season and at harvest, based on discussions with farmers during the early phases of participatory research in Kenya, (De Groote and Siambi, 2005). Typically, participants are shown only one replication of the trial, containing one variety per plot, to limit the number of plots to be evaluate by each participant to less than 15-20. Preferably, the evaluation is double blind, where neither the participating farmers nor the enumerators know which variety is in a particular plot. It is advisable to randomize the order in which plots are evaluated to minimize order, fatigue and learning effects (Carlsson et al., 2012; Czajkowski et al., 2014).

#### 2.4.2 Empirical framework and tools

The common tool for farmer evaluation of the new hybrids was a short questionnaire that could be filled in by participating farmers themselves with the help of trained enumerators (Supplementary material S1). The questionnaire consisted of three components: socioeconomic characteristics, plant traits, and overall performance evaluation. The first component included questions about consent to proceed with the interview, location of the trial, socioeconomic characteristics of the respondent such as age, gender, education (number of years in formal education and highest level attained), wealth of the household (income and land owned), and maize production characteristics (including area of maize, adoption of fertilizer and improved varieties). The second component of the questionnaire was a table with a list of traits identified earlier in Kenya (De Groote et al., 2002). For each trait, participants were asked to quantify its importance on a scale of 0 (not important) to 3 (very important) (see table on the fourth page of the questionnaire, Supplementary Material S1). These values can be considered indicators of the farmer-stated preferences for different criteria. The third component was a continuation of the same table, with one column for each variety, identified by the plot number in the column head, but not by name. The table had a row for each trait, and the participant was asked to score each variety on a five-point Likert scale. To avoid the ambiguity of numerical scores (where 1 can be interpreted as first or 5 as the highest), we used letters, where A = like strongly, B = like, C = neither like nor dislike, D = dislike, E = dislike strongly. In the final row of the table, participants were asked to score the variety for overall performance using the same scale.

#### 2.4.3 Study design

Because of budget constraints, participatory evaluations could only be conducted in Kenya and Rwanda. To establish the possible sites for evaluations, reconnaissance visits were conducted to all sites in these countries by a team of breeders and social scientists, using the criteria outlined in the previous section. In the early-to-intermediate maturity set, 13 out of the possible 42 environments were randomly selected, while for the intermediate-to-late maturing hybrids set, 14 out of the possible 40 environments were randomly drawn. Over the two years of the study, participatory evaluations were undertaken at twelve sites in Kenya and two sites in Rwanda covering both sets (Fig. 1).

Farmer mobilization was carried out by the local collaborating institutions: Kenya Agricultural and Livestock Research Organization (KALRO) at Embu, Kakamega and Katumani stations in Kenya, and the Rwanda Research Board (RAB) in Rwanda. At each site, the target was to have at least 60 farmers for each group of varieties, and farmers from the area were invited through the local administration, extension officers, and farmer groups. Usually, between 120 and 200 farmers would attend a farmer field day at a given site (Table 2). Enumerators were recruited with the assistance of officers from the collaborating institutions who had a background in agriculture and were familiar with the local language. Enumerators were trained in the use of the questionnaire, which was adapted from previous farmer evaluations (De Groote et al., 2002; Neubauer et al., 2018) and contained the three major sections discussed above: socioeconomic variables, importance of criteria, and evaluation of the varieties.

#### 2.4.4 Data collection

On arrival, farmers were registered and allocated alternately to either the early-to-intermediate or the intermediate-to-late maturity set. Next, the first and second sections of the questionnaire that contained the socioeconomic questions and the list of traits were administered individually. For the third section, a facilitator first outlined the next steps of the variety evaluation and translated the meaning of each trait into local context and language so that the participants could understand what it was. Further, the facilitator explained how to use the five-level Likert scoring scale from strongly dislike to strongly like. The participants were then divided into groups of 5–10 farmers per group and given an enumerator to guide them in the scoring from one plot to another. The scoring was strictly individual, however, while participants were encouraged to discuss the different varieties and their traits, they were discouraged to exchange their scores or read each others’ score sheets.

Each plot was marked on the four corners with an entry number of the hybrid/variety in that plot (Table 1). This number also corresponded with a column on the questionnaire. Farmers would then congregate after the evaluation, and the questionnaires would be collected by the team and checked for completeness. A total of 2025 farmers took part in the evaluations: 462 participants in 2016 and 1563 in 2017 (Table 2). More women than men attended: 54% percent of participants over the two years were women.

#### 2.4.5 Analysis of participatory evaluation

The data collected were analyzed using SPSS (Statistical Package for the Social Sciences, Version 24.0. Armonk, NY: IBM Corp.) and STATA (Statistical/Data Analysis, Version 14.0, College Station, Texas: StataCorp). Means and descriptive statistics were used to analyze a participant’s socioeconomic characteristics. Means were also used to analyze the importance of various criteria, given the weights that participants stated for each criterion.

The data obtained from farmers’ evaluations were scores, which were ordered as categorical data. The preferred analysis of such data is ordinal regression (McCullagh, 1980). Therefore, to analyze the different hybrids in the trials, we used ordinal regression with the scores, for the different traits and overall, as the dependent variables (Coe, 2002; De Groote et al., 2010). To evaluate how the socioeconomic characteristics of participants such as gender, education and wealth affected the preferences of the respondents, these characteristics were entered as interactions with the hybrids (varieties) (De Groote et al., 2010, 2014).

To analyze how the performance of varieties for different traits affected a farmer’s overall evaluation, we used ordinary least squares (OLS) regression with, for each variety, its overall score as a dependent variable and its scores for individual traits as explanatory variables. The coefficients obtained for each trait account for the weight of this trait to the participants overall (De Groote and Siambi, 2005). Finally, the results from the participatory evaluations were compared with on-farm grain yield results from the same hybrids, averaged over the participatory evaluation sites.

## 3 Results

### 3.1 Grain yield performance and genotype by management level interaction

Analysis of variance for grain yield showed significant differences (*P* <  0.05) among the early-to-intermediate maturity set of hybrids for both management levels (<3 and >3 t ha^−1^) (Table 3). Combined analysis also revealed that variances due to genotypes, site, management, and genotype × management were highly significant (*P* <  0.01). The presence of highly significant genotype × management interactions indicated differential performance of the hybrids under different management levels. For the intermediate-to-late maturity set, there were highly significant differences (*P* <  0.01) among the hybrids under optimal management level (Table 4). Heritability estimates ranged from 0.41 to 0.89 for both sets of hybrids (Table 3, Table 4).

**Table 3:** On-farm grain yield (t ha^−1^) performance of early-to-intermediate hybrids under low yielding environments (<3 t ha-1), high yielding environments (>3 t ha-1), and across 42 testing environments, 2016–2017.

Entry	Hybrid/Variety	Yield under	Yield under	Yield t ha^−1^
		>3 t ha^−1^ environment	<3 t ha^−1^ environment	Combined
1	CKH122114	6.22	1.42	3.82
2	EMH1101	5.31	1.59	3.45
3	CKH143975	6.91	1.93	4.42
4	WE3102	6.33	1.63	3.98
5	WE4120	6.33	1.78	4.06
6	WE3106	6.04	1.64	3.84
7	WE4109	5.69	1.42	3.56
8	FARMER CHECK	5.09	1.17	3.13
9	WE3101 (IC)	5.71	2.02	3.87
10	DLSH103 (IC)	6.19	1.21	3.7
11	DUMA43 (CC)	5.01	0.95	2.98
12	PAN4M-19 (CC)	4.4	1.22	2.81
	Heritability	0.89	0.51	0.65
	Genotype	14.53**	1.34*	7.98**
	Site	40.41**	15.60**	32.96**
	Management			74.60**
	Genotype × Management			2.83**
	Residual	1.51	0.67	1.26
	Grand mean	5.77	1.5	3.63
	LSD (5 %)	0.65	0.65	0.53
	CV (%)	5.6	21.62	7.33

**Table 4:** On-farm grain yield (t ha-1) performance of intermediate-to-late maturity hybrids under low yielding environments (< 3 t ha-1), high yielding environments (>3 t ha-1), and across 40 testing environments, 2016–2017.

Entry	Hybrid	Yield under	Yield under	Yield t ha^−1^
		>3 t ha^−1^ environment	<3 t ha^−1^ environment	Combined
1	CKH10769 (IC)	6.14	1.12	3.63
2	CKH13605	5.65	0.86	3.25
3	CKH143770	6.03	2.18	4.1
4	CZH0837 (IC)	5.75	1.29	3.52
5	FARMER CHECK	4.47	1.16	2.81
6	KM1201	5.89	1.24	3.57
7	PHB30G19 (CC)	5.54	1.07	3.31
8	WE1101 (IC)	5.67	1.25	3.46
9	WE2106	5.62	1.48	3.55
10	WE3104	5.86	0.73	3.3
11	WE3105	6.03	1.24	3.63
12	WH505 (CC)	5.54	1.37	3.45
13	WH509 (CC)	5.51	1.33	3.42
	Heritability	0.67	0.41	0.29
	Genotype	5.38**	0.97	2.24
	Site	36.81**	11.43**	31.47**
	Management			82.35**
	Genotype × Management			1.7
	Residual	1.71	0.58	1.48
	Grand mean	5.67	1.26	3.46
	LSD (5%)	0.66	0.73	0.66
	CV (%)	5.86	28.95	9.51

Grain yield ranged from 4.40 to 6.91 t ha^−1^ and from 0.95 to 2.02 t ha^−1^ under optimal management and random stress farmers’ conditions, respectively, for the early-to-intermediate maturity set (Table 3). An identical trend in grain yield was also observed with respect to the two management levels for the intermediate-to-late maturity set (Table 4). The mean grain yield of the hybrids across the environments (site-management-year combinations) ranged from 2.81 to 4.42 t ha^−1^ (mean of 42 environments) and 2.81 to 4.10 t ha^−1^ (mean of 40 environments) in the early-to-intermediate and intermediate-to-late maturity sets, respectively.

Among early-to-intermediate maturing hybrids, the top three hybrids (CKH143975, WE4120 and WE3106) produced a 45.9–65.6% higher grain yield than the best commercial check, PAN4M-19, under low-yielding environments (Table 3). Under optimal testing environments, the top three new hybrids (CKH143975, WE4120 and WE3102) produced 26.3–37.9% higher grain yield than the best commercial check, DUMA43. Among the intermediate-to-late maturing hybrids, the best hybrid (CKH143770) produced a 59.1% higher grain yield than the best commercial check, WH505, in low-yielding environments (Table 4). The top three new hybrids among the intermediate-to-late maturing set (CKH143770, WE3105 and KM1201) produced a 5.8–8.8% higher grain yield than the best commercial check WH505 in an optimal environment. The most recently developed hybrids, CKH143975 (early-to-intermediate) and CKH143770 (intermediate-to-late) outperformed the internal genetic-gain checks in the respective maturity sets across environments (Table 3, Table 4).

The results of pair-wise comparison between the new stress-tolerant pre-commercial hybrids and the best commercial checks showed that the highest-yielding pre-commercial hybrid among the early-to-intermediate maturity set (CKH143975) had a 1.4 t ha^−1^ yield advantage over the best commercial check (LSD = 0.53, see Table 3 and Supplementary Table A1). Similarly, the highest-yielding pre-commercial hybrid in the intermediate-to-late maturing set (CKH143770) had a 0.7 t ha^−1^ yield advantage over the best commercial check (LSD = 0.066, see Table 4 and Supplementary Table A2).

The adaptability and performance of the hybrids across the low yield and optimal environments over the two years are presented in Supplementary Figs. S1 and S2. The hybrids that are in the same direction to one environment have a good performance in that environment, and hybrids with high value of the first principal component score and near zero value for the second principal component score were the most stable with the highest yield performance on those environments. For the early-to-intermediate maturity set, the first component explained 90.6% of the total variance (Supplementary Fig. S1). One stress-tolerant (ST) CIMMYT maize hybrid, CKH143975, was a stable genotype as it had a positive first principal component (PC1) score and close to 0 s principal component (PC2) score. Some ST hybrids performed relatively better either under stress or under optimal conditions. For example, hybrid WE3101 followed by WE4120 performed best under stress conditions compared to the other hybrids (Supplementary Fig. S1). On the other hand, hybrids WE3102 and CKH12114 performed best under optimal conditions. Principal component 1 explained 62.3% of the total variance for the intermediate-to-late maturing set (Supplementary Fig. S2). Stress-tolerant hybrid CKH143770 showed good performance under stress conditions, while hybrids CKH10769 and WE3105 were the best under optimal conditions (Supplementary Fig. S2). All commercial checks in both maturity sets were in the opposite direction in both testing environments, indicating that the new ST hybrids performed better across the two management levels compared to the checks. However, the top yielding varieties for stress and optimal conditions were different.

### 3.2 Participatory evaluation of new maize hybrids

#### 3.2.1 Characteristics of the participants

In total, 2025 farmers participated in the on-farm variety evaluations, in two countries over two years. More than half of the participants (54%) were women. Participants had a mean age of 42 years. The average number of years of formal education was nine, although this was less in Rwanda (five). Almost all participants were farmers, with an average experience in farming of 16 years, which was higher in Rwanda (20 years). The mean household income reported was 836 USD and the mean land holding was two acres per household (Table 5). Participants had high adoption levels for improved maize varieties, with the highest level in Rwanda (98%) and the lowest in eastern (86%) (Table 5).

**Table 5:** Socioeconomic characteristics of 2025 farmers that participated in the on-farm variety evaluation.

		Kenya						Rwanda		Total
	Characteristic	Central mid-altitude	Eastern mid-altitude	Eastern transitional	Western mid-altitude	All		Mid altitude		
Participants PVE	Number (total)	404	345	123	975	1847		178		2025
	Women (%) total	66	63	41	49	55		45		54
Adoption	Improved maize varieties (%)	91	86	87	91	87		98		91
	Inorganic fertilizers (%)	85	87	91	89	87		99		89
Age (years)	Mean	38.72	35.27	50.23	45.08	43.51		43.09		42.18
	Std. Deviation	14.90	13.83	15.79	15.66	16.92		12.76		15.58
Farming Experience in years	Mean	12.19	11.17	22.47	17.58	18.05		20.10		15.84
	Std. Deviation	11.58	11.07	14.89	13.08	17.50		14.38		13.15
Years of formal education	Mean	9.46	10.77	11.84	9.88	10.16		6.32		9.71
	Std. Deviation	4.09	7.16	13.02	12.42	11.28		9.29		10.22
Area of farm owned (acres)	Mean	1.50	1.43	4.22	3.86	3.22		2.69		2.87
	Std. Deviation	4.60	1.38	11.36	11.77	10.74		8.57		9.25
Mean area under maize (acres)	Mean	0.78	0.70	1.50	1.22	2.68		1.64		1.09
	Std. Deviation	0.78	0.79	1.88	1.05	2.12		4.57		1.71
Total income (USD/household/year)	Mean	987	920	3378	692	1097		1228		1000
	Std. Deviation	2708	1513	27004	1252	8259		2962		6908
Area under maize in (ha)										
Female	Mean	0.43	0.29	0.56	1.25	0.91		0.78		0.78
	Std. Deviation	5.40	0.71	2.36	12.83	10.53		3.90		8.92
Male	Mean	0.37	0.29	1.66	1.09	1.06		0.97		0.91
	Std. Deviation	0.86	0.91	14.27	10.48	10.74		10.09		9.25
Total	Mean	0.41	0.29	1.22	1.17	0.98		0.89		0.84
	Std. Deviation	4.41	0.79	11.19	11.68	10.62		7.91		9.08
Own farm size in (ha)										
Female	Mean	0.61	0.60	0.91	1.64	1.23		1.45		1.12
	Std. Deviation	5.57	1.34	3.01	12.89	10.62		11.34		9.48
Male	Mean	0.65	0.59	2.33	1.57	1.49		0.85		1.28
	Std. Deviation	1.54	1.45	14.35	10.62	10.88		5.32		8.98

#### 3.2.2 Farmer-stated trait preferences for the evaluation of maize hybrids

**Fig. 2:**
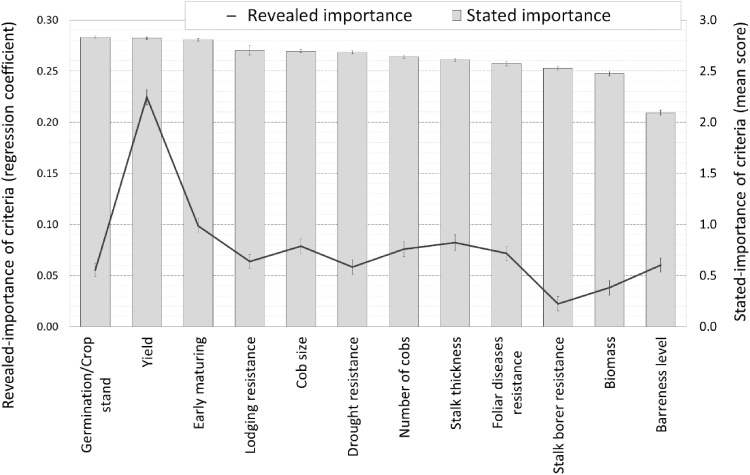
Farmer-stated vs farmer-revealed criteria of importance in the evaluation of maize hybrids evaluated across different management conditions in East Africa from 2016 to 2017 (error bars represent standard errors).

Farmers were asked to state the importance of different traits of maize hybrids, using a score of 0 (not important) to 3 (very important). We can call this the stated-importance of criteria or the stated preferences. The three most important criteria they mentioned were early maturity, germination (an indicator of quality of seed if the soil moisture is sufficient), and grain yield, all of which received an average importance score of 2.8 (Fig. 2, stated criteria). Other important criteria (all receiving a score of 2.6–2.7) were good husk cover, cob size, drought tolerance, and number of cobs. In the next group of criteria (with scores between 2.5 and 2.6) were resistance to lodging and the related stalk thickness, and resistance to diseases and insect-pests. In the stated-importance criteria, there were no significant differences in importance of traits between men and women.

#### 3.2.3 Trait preferences revealed by farmers for the evaluation of maize hybrids

To estimate the revealed preferences for traits, as compared to the stated preferences, we regressed the overall scores for the hybrids on the scores for the individual traits. The regression coefficients for all the traits were significant, indicating that they all played a role in determining the overall score (Table 6). Further, the coefficients added up to almost 1 (0.97), so the coefficient for each trait could be interpreted as an indicator of the weight of that trait, which we could call the revealed preference or the revealed importance of the trait. From this analysis, we found that yield was the most important trait, with a coefficient of 0.23, indicating that an increase in yield score of one unit led to a 23 percent rise in overall appreciation of the hybrid in question. Early maturity was the second most important trait (0.10), followed by stalk thickness, cob size and number of cobs (all three 0.08), resistance to disease, and germination (both 0.07).

**Table 6:** Revealed preferences, obtained by regressing the overall evaluation score on the evaluation scores of individual criteria.

Individual criteria	Coef.	Std. Err.	P>t
Constant	−0.0350	0.0186	0.060
Yield	0.2295	0.0075	0.000
Early maturing	0.0993	0.0069	0.000
Cob size	0.0880	0.0073	0.000
Number of cobs	0.0826	0.0075	0.000
Foliar diseases resistance	0.0724	0.0071	0.000
Germination/Crop stand	0.0707	0.0066	0.000
Lodging resistance	0.0662	0.0068	0.000
Barrenness level	0.0657	0.0071	0.000
Drought resistance	0.0611	0.0074	0.000
Biomass	0.0481	0.0073	0.000
Cob rot resistance	0.0458	0.0069	0.000
Stalk borer resistance	0.0357	0.0072	0.000
Number of observations	14905		
R-squared	0.6294		
Root MSE	0.69286		

There is a clear contrast between the stated- and revealed-importance of criteria. In farmer-stated preferences, farmers mention a large number of traits, and give many of them the highest importance. Three traits receive an average importance score of 2.8 (out of a maximum of 3), while seven more receive an importance score higher than 2.5. The farmer-revealed preferences, on the other hand, provide more nuance, with yield standing out as having by far the most importance (regression coefficient or weight of 0.22), and early maturity as the second most important criteria (0.10). Only after those two a range of other criteria follow with similar importance.

**Fig. 3:**
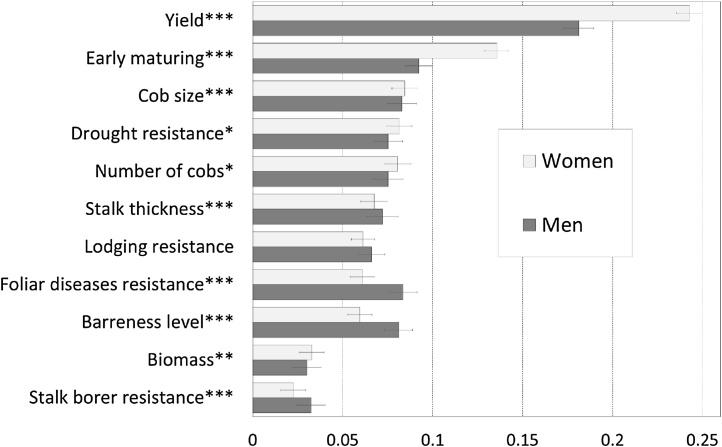
Revealed criteria contribution to overall evaluation by gender (asterix indicate significant differences between men and women, error bars are standard errors of the regression coefficients).

There were significant differences (*P* <  0.1) between men and women in the revealed trait preferences (criteria) for all traits except for resistance to lodging. However, both women and men showed a higher preference for yield and early maturity (Fig. 3). Comparing the stated- with the revealed-importance preferences (Fig. 2), we found out that in revealed-importance preferences, yield and earliness were the most important criteria for both men and women farmers (Fig. 3).

#### 3.2.4 Farmer evaluation of the different hybrids

**Fig. 4:**
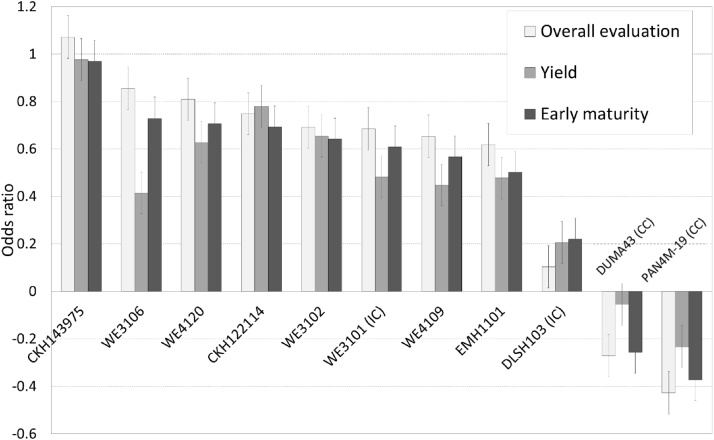
Farmer participatory evaluation of early-to-intermediate variety performance (results of ordinal regression analysis, omitted category is farmers' check, error bars are standard errors).

To analyze farmers’ evaluation of the different hybrids, we first used the overall score. As the most important criteria in both the stated and the revealed analysis were yield and early maturity, we also analyzed the scores for those criteria by maturity set. In the early-to-intermediate maturity set, the new improved hybrids were generally much better appreciated by farmers than the commercial and the farmers' checks (omitted category), for overall performance and for the most important criteria: yield and early maturity (Fig. 4). The best hybrid for all three criteria was CKH143975. The second-best hybrid, WE3106, was well appreciated overall and for early maturity, but not for yield. All the new hybrids were better appreciated than all the checks, except for the internal genetic gain check, WE3101, which was also much appreciated and came out sixth, ahead of two new pre-commercial hybrids.

**Fig. 5:**
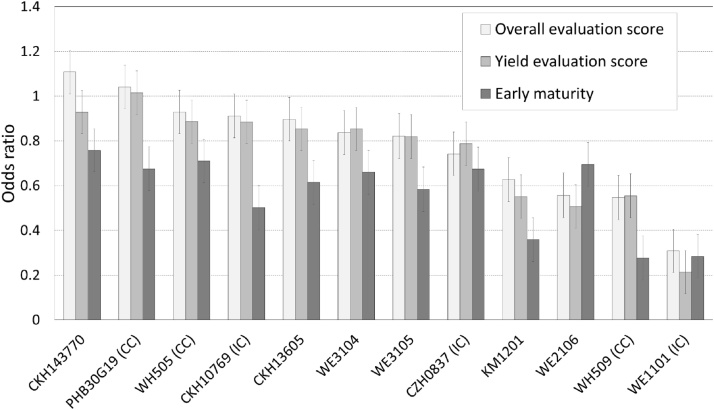
Farmer evaluation of intermediate-to-late varieties (numbers are coefficients of the ordinal regression, which are odds ratios, with farmers' check as omitted category, error bars are standard errors).

Among the intermediate-to-late maturing hybrids, all the new improved hybrids were better appreciated than the farmers' check, the omitted category in the ordinal regression (Fig. 5). However, they did not generally outperform the commercial checks. Still, the best hybrid was the new hybrid CKH143770. Second and third performers, however, were the commercial checks PHB30G19 and WH505, followed by the internal check CKH10769. In terms of farmers’ yield score, the commercial check PHB30G19 was the best variety. There were some differences in farmer evaluations between management levels. For overall evaluation scores, varieties EM1101 and WE4120 did better under marginal conditions, while others (WE1102, WE3101, Pan4m19) did better under optimal conditions and poorly under marginal conditions. The rest of the varieties (WE4120, DLSH103 and WE4109) performed equally well in both environments. Unlike for the early-to-intermediate maturing hybrids, where participatory evaluation was conducted under all management levels, participatory evaluations for the intermediate-to-late maturity set were conducted only in the optimal management sites (yield >3 t ha^−1^), so comparison between management levels was not possible for the intermediate-to-late maturing set.

#### 3.2.5 Comparison of farmer evaluation score and on-farm grain yield performance

**Fig. 6:**
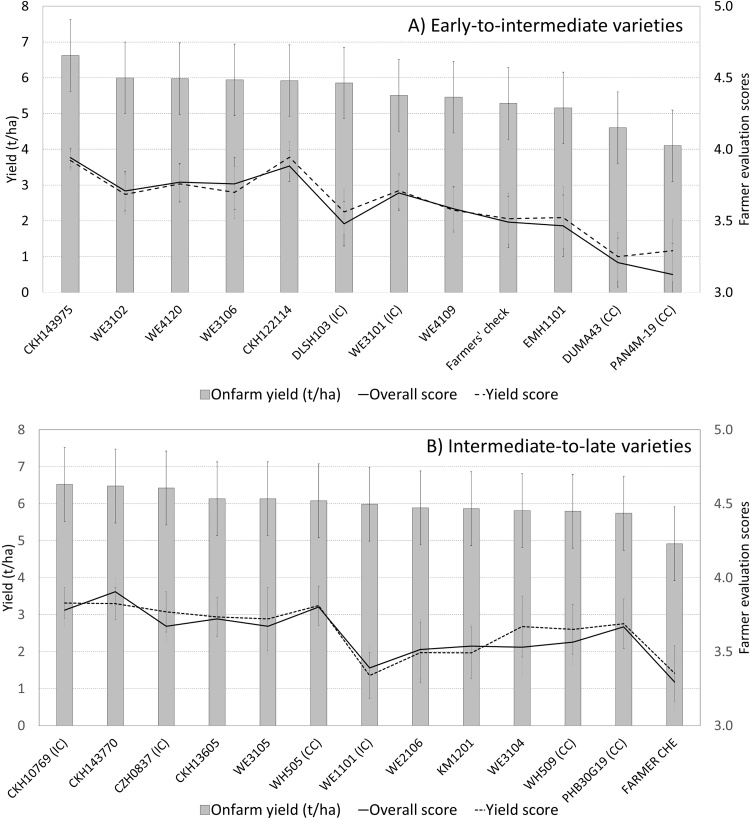
Comparing breeders' (yield) and farmers' evaluation (average evaluation scores for overall and for yield) (error bars are standard errors).

Finally, we compared the farmers’ evaluation, as per the scores they gave, to the on-farm grain yield performance (Fig. 6, Panel A). To make proper comparison possible, for yield analysis we only included the sites where Participatory Variety Evaluations (PVEs) with farmers were conducted. In the early-to-intermediate hybrids set, CKH143975 came first, as it had the highest yield as well as the highest overall score and yield score by farmers. However, farmers valued WE3106 as second best, although yield-wise it was ranked fourth (although, admittedly, the yields of the 2^nd^ to 6^th^ best hybrids were very close). Another discrepancy is the 6^th^ hybrid, DLSH103, that had a good yield, but received low scores in farmers’ evaluations, for overall as well as yield. This variety scored below average for most traits, but most notably the plant height and cob related aspects (husk cover, cob size, barrenness level and number of cobs).

In the intermediate-to-late maturing group (Fig. 6, Panel B), we also saw large discrepancies between yields and farmers' evaluations. However, farmers top-preferred hybrid was CKH143770, which was similar to the yield evaluation. Farmers second most-preferred hybrid was the commercial check, PHB30G19, which came next to last in terms of yield (just before the farmer check). PHB30G19 scored better than average on moist traits, in particular on height, cob size and lodging resistance. Similarly, WH505 was not among the top yielding varieties, but it received better scores than average for cob size, stalk thickness, number of cobs and height. In contrast, two varieties had good yields but received low scores on farmer evaluations: WE1101 scored below average on all traits, but especially on cob size, yield, stalk thickness, height and good husk cover, while WE2106 scored below average on most traits, but especially on barreness level and good husk cover.

## 4 Discussion

In this study, we assessed the grain-yield improvements made in early-to-intermediate and intermediate-to-late maturing CIMMYT hybrids developed from 2010 to 2014 under on-farm conditions, and asked farmers to evaluate them. The hybrids were selected from different years, based on data from on-station, optimal, managed stress and random stress conditions (Beyene, Mugo et al., 2013; Makumbi et al., 2018; Worku et al., 2016).

In the early-to-intermediate maturity set, the top three new ST hybrids CKH143975, WE4120 and WE3102 outperformed the best commercial check under both low yielding (<3 t ha^−1^) and optimal yielding (>3 t ha^−1^) management. The new ST hybrids showed a higher yield advantage over the commercial check under stress conditions than under optimal conditions. A similar yield-advantage trend was observed for the best hybrid in the intermediate-to-late maturity set (CKH143770), with a more pronounced yield advantage of the new ST hybrids under stress conditions than under optimal conditions. The on-farm yield advantage of CIMMYT’s stress-tolerant hybrids over commercial checks was also documented earlier: Setimela et al. (2017) tested hybrids developed in the 2000s and reported an on-farm yield gain of 4–19% over popular commercial checks; while under severe drought- and heat-stress conditions during the 2015/2016 El Niño year in southern Africa, a 20 % grain yield advantage of ST hybrids over the commercial checks was reported (Setimela et al., 2018) The results substantiate the success of CIMMYT’s breeding approach for increasing maize productivity under the stress conditions experienced in diverse African smallholder farmers’ conditions (Ceccarelli et al., 1992; Bänziger et al., 1999).

For any breeding program it is important to assessing the improvements and monitor the efficiency of the breeding program (Masuka et al., 2017). In the early-to-intermediate maturity set we found that the across mean grain yield of the best ST hybrids, WE4120 (developed in 2014) and CKH143975 had yield advantages of 4.9% and 14%, respectively, over the across mean grain yield of the best internal check, WE3101 (developed in 2013). In the intermediate-to-late maturity set, the best ST hybrid, CKH143770 (developed in 2014) also had across mean grain yield advantage of 16.5% over the internal genetic check CZH0837 (developed in 2008). These results implied good on-farm yield gain under farmers’ conditions in both the early-to-intermediate and intermediate-to-late maturity groups. Setimela et al. (2017) reported that CZH0837 was among the best ST hybrids in on-farm trials in ESA from 2010 to 2011. However, in the current study, CZH0837 was out-yielded by the recently developed hybrids, revealing progress in increasing genetic gain under the diverse management environments of eastern Africa.

The best new hybrids (CKH143975 in the early-to-intermediate set, and CKH143770 in the intermediate-to-late maturity set) outperformed the best commercial checks by more than 18%, and the best internal checks by 12–14%, and have been recently recommended for release in Uganda and Kenya, respectively. Among the internal genetic-gain checks, WE3106, CKH10769 and WE1101 have already been commercialized in Kenya and Uganda, while CZH0837 has been commercialized in Ethiopia and Tanzania. DLSH103 is also commercialized in Kenya.

The SREG biplot model showed a distinct pattern of adaptation of the hybrids, and stability across the testing environments (Supplementary Figures A1 and A2). Most of the ST hybrids in both maturity groups were positioned to the right side of the biplot, with the two management environments (<3 and >3 t ha^−1^) revealing better performance of these hybrids under both management conditions, compared to the hybrids positioned on the opposite direction of the biplot (Torres Flores et al., 2017). All the commercial checks in both maturity groups were positioned on the left side of the biplot, indicating that the new ST hybrids performed better under the diverse production environments of eastern Africa, compared to the commercial checks.

Based on the analysis of farmers’ overall scores vis-à-vis their scores for individual traits, yield came out as the most important trait, followed by early maturity, cob size and number of cobs, and a range of minor, but still significant indicators. As a result, farmers evaluate the hybrids on a wider range of criteria than yield and give those other criteria more weight than breeders may tend to do. This difference has been frequently observed in previous PVS research, for example in different crops in India (Joshi and Witcombe, 1998), in barley in Morocco (Ceccarelli and Grando, 2007) and also in maize in Kenya (De Groote et al., 2002; Neubauer et al., 2018).

It is useful to consider farmers’ criteria in the stage-gate advancement process of new hybrids by the breeders, irrespective of whether or not the farmers’ evaluation scores are similar or dissimilar to breeders’ evaluations. In both early-to-intermediate and intermediate-to-late maturing hybrids, for example, the scores of the top hybrids based on yield and on farmer evaluation were the same. However, there were some important discrepancies among other hybrids. Farmers did not like some high-yielding hybrids, but on the other hand scored some lower-yielding hybrids quite highly. We do not have a good understanding where such discrepancy comes from, but it needs further investigation. We speculate that the different methods used by maize breeders and farmers may account for such observations. Maize breeders estimate grain yield through measurements while farmers usually apply visual scores. That farmers score some varieties low on yield, while their measured yield was average to good, is likely related to the poor performance of these varieties on other traits, especially cob-related aspects, which affects farmers’ perception of their yield. Kernel depth, kernel density and grain moisture content can easily lead to farmers missing actual grain yield in their scores unless they measure the actual grain yield. Further, our results showed that the yields of some improved hybrids do not differ that much, especially in the intermediate-to-late maturity set. Farmers evaluations, on the other hand, varied much more widely. Hence, a farmer will look at traits other than yield, and those traits will be increasingly important in successfully marketing new hybrids, especially if their yields do not differ much.

An interesting approach to evaluate the relative importance of different farmer criteria was to first ask farmers to indicate the importance of those criteria. When the question is phrased this way, the results is a large number of traits, and almost all are scored as “very important”. However, when the importance of those traits is revealed by regressing the varieties’ scores for individual traits on the overall score, the results are more nuanced. Further, analyzing the traits on which good-yielding varieties score poorly under farmer evaluations provides interesting insights on how to improve these varieties.

In this study, we tried to analyze whether there were major differences in preferences for traits as well as for individual hybrids among different groups of farmers, in particular by gender, age and wealth. We found statistical differences between men and women in terms of the importance of criteria; women gave relatively more importance to yield and early maturity, while men gave more importance to resistance to foliar diseases and barrenness levels. However, this did not alter the relative importance between criteria, which were very similar between men and women and the evaluations of the different hybrids did not differ between men and women. Similarly, little or no differences were observed between age and wealth categories. This homogeneity of farmer’s field evaluations confirms previous research (Florian et al., 2018) and prior expectations: intuitively, at this level, we would not expect much difference. Where men and women do differ, is in the importance of post-harvest and processing characteristics, which are more important for women.

## 5 Conclusion

The results of this study confirmed that CIMMYT-derived ST maize hybrids have a much better grain yield performance than the best commercial checks in the eastern Africa region under stress environments of smallholder farmers but have a comparable grain yield performance under optimal conditions. ST hybrids also showed better grain-yield stability across the testing environments, providing evidence for the success of the CIMMYT maize breeding approach (increasing maize productivity under stresses experienced in diverse African agro-ecologies and smallholder farmers’ conditions). In addition, the new ST hybrids outperformed the internal genetic checks, indicating continued genetic gain under farmers’ conditions. The results of this study also showed that the top-yielding hybrids in both maturity groups gained the highest farmers’ overall scores, revealing the ability of smallholder farmers to select suitable hybrids for their own conditions. Farmers gave high score to grain yield in both farmer-stated preferences and farmer-revealed preferences of traits. The evaluations of the different hybrids did not differ between men and women. However, grain yield performance and farmers’ overall score differed for some hybrids, indicating the need for further investigation. Farmers gave priority to different traits in addition to grain yield, but these were specific to the environments under which selection was done and may not be applicable across all maize-growing environments. Farmers-stated importance of the different traits were different from farmer-revealed importance, as derived from regressing the overall scores on the scores for the individual criteria. As the revealed importance provided more nuance, this approach can be recommended for future work, in combination with analysis of the traits on which good-yielding varieties receive poor scores during farmer evaluations. We conclude that incorporating farmers’ selection criteria and farmer evaluation in the development of new maize hybrids is important under the changing maize-growing environments in sub-Saharan Africa, and recommended to increase the varietal turnover of maize hybrids and improve the food security in the region.

## Declaration of Competing Interest

None.
