# Supplementary material for: On-farm performance and farmers’ participatory assessment of new stress-tolerant maize hybrids in Eastern Africa
Source: Field Crops Res. 2020 Feb 1;246:107693. doi: 10.1016/j.fcr.2019.107693 (PMC6961973; doi:10.1016/j.fcr.2019.107693)
Supplement: Supplementary file 1 [file mmc1.docx]

**Supplementary materials**

**Supplementary material S1. Questionnaire**

Part 1 . **Introduction and Informed Consent**

Enumerator Name: ________________________________Enumerator Code: __________

Good morning/Afternoon. My name is ______ I am coming from International Maize and Wheat Improvement Center (CIMMYT). CIMMYT is currently working Participatory farmer evaluation of maize varieties for the IMAS project”. The primary goal of this evaluation is to understand farmers’ appreciation of various traits as expressed by improved varieties.

This site has been randomly selected from sites where regional variety trials are taking place in Kenya and you the farmers from the area were invited to participate in this exercise. You were not selected for any specific reason and your participation is voluntary.

I would like to ask the questions from this form to you as farmer, and thereafter you shall participate in evaluations for trials in the farm. The whole exercise will take about 90 minutes to complete. All your answers will be held in confidence. The answers which you might give me will only be used by CIMMYT or under CIMMYT’s supervision.

Before I start, do you have any question or is there anything that I have said for which you would like further clarifications? May I proceed with interviewing you? _____Yes _____No ________Initials

Should you have any question or concern, please contact Dr. Hugo De Groote (0722595165), Michael Ndegwa (0720269326) or Bernard Munyua (0722401489)

RESPONDENT IDENTIFICATION AND CHARACTERISTICS/MASWALI YA KUJITAMBULISHA

1. Date of the evaluation/ Tarehe: _________/________/________
2. District/Sub-County________________ Division/Ward_____________________
3. Location/ Kata_________________ Sub location/Kata ndogo_____________
4. Participant’s name/ Majina ya mhusika ______________________________________
5. Mobile number/Nambari ya simu___________________________________________
6. Gender of the participant/ Jinsia ya mhusika: Male/Mme=1, female/Mke=0 _________
7. Age/Umri: ______(years/miaka), Experience in farming/miaka ya ukulima _________
8. Years of formal education/miaka ya elimu rasmi: ______________________________
9. Highest level of education attained/kiwango cha juu ya elimu ulichofika: ___________

(*Lower primary/shule ya msingi hadi darasa la nne =1, Upper primary school/ shule ya msingi hadi darasa la nane=2, secondary/shule ya upili=3, Tertiary/elimu ya juu=4)*

1. Size of the farm (acres)/ekari za shamba lako: _____ _____________________
2. Area under maize (acres)/ekari zilozopandwa mahindi: ________________________
3. Did you purchase improved maize seed this season/Ulinunua mbegu za mahindi za pakiti huu msimu? (0=NO/La, 1=Yes/Ndio) ______________________________________________

If yes which were the three-major certified maize variety planted (be specific)

MZVAR1______________________

MZVAR2______________________

MZVAR3______________________

1. Did you use chemical/fertilizer this season/ulitumia mbolea ya nduka kupandia huu msimu?_______

(0=NO/La, 1=Yes/Ndio) ___________________

1. Has your household had contact with an extension agent (field days, demo/office/farm visit) in the last one year? (0=NO/La, 1=Yes/Ndio)___________
2. Category/type of participant/Aina ya mhusika: (check one/weka alama kwa moja)

| **Respondents category/ Aina ya mhusika** | **Tick one/ Chagua moja** |
| --- | --- |
| 1. Farmer who hosts the trial/Mkulima aliyepanda mahindi ya utafiti |  |
| 1. Invited farmer/ Mkulima aliye alikwa |  |
| 1. Extension officer/Afisa wa kilimo |  |
| 1. Administration officer/Afisa wa utawala |  |
| 1. Other participant/Mwingine :______________________ |  |

1. Does your household have a **Mobile** phone (0=NO/La, 1=Yes/Ndio)_________________
2. Does your household have a **Bicycle** the household (0=NO/La, 1=Yes/Ndio)___________
3. Does your household have a **Bank account** (0=NO/La, 1=Yes/Ndio)_________________
4. Cash household income last year (January to December 2017)? Ksh_________________

(Consider cash from crop, livestock sales, salaries wages and remittances)

**Part 2:** Enumerator Name: ________________Enum Code: __________Please indicate the plot that you **started** **with**. **STARTPLOT**__________ **A** = like very much/Napenda sana, **B**= like/Napenda, **C**= neither like nor dislike/niko hapo katikati tu, **D**= dislike/Sipendi, **E**= dislike very much/Sipendi kabisa

| **Criteria/ Mizani** | **How Important is this criteria**? |  | **Plot 1** | **Plot 2** | **Plot 3** | **Plot 4** | **Plot 5** | **Plot 6** |  | **Plot 7** | **Plot 8** | **Plot 9** | **Plot 10** | **Plot 11** | **Plot 12** |
| --- | --- | --- | --- | --- | --- | --- | --- | --- | --- | --- | --- | --- | --- | --- | --- |
| 1. Germination/Crop stand |  |  |  |  |  |  |  |  |  |  |  |  |  |  |  |
| 1. Height/urefu wa mmea |  |  |  |  |  |  |  |  |  |  |  |  |  |  |  |
| 1. Stalk thickness/ unono wa mti |  |  |  |  |  |  |  |  |  |  |  |  |  |  |  |
| 1. Resistance to stalk borer/ Kustahimili **stalk borer** |  |  |  |  |  |  |  |  |  |  |  |  |  |  |  |
| 1. Biomass (for fodder)/ wingi wa majani ya lishe |  |  |  |  |  |  |  |  |  |  |  |  |  |  |  |
| 1. Barrenness level/ kiwango cha utasa wa mmea |  |  |  |  |  |  |  |  |  |  |  |  |  |  |  |
| 1. Number of cobs per plant/numbari ya **misakwa** kwa mmea mmoja |  |  |  |  |  |  |  |  |  |  |  |  |  |  |  |
| 1. Cob size/ ukubwa wa **misakwa** |  |  |  |  |  |  |  |  |  |  |  |  |  |  |  |
| 1. Good cover of the husk/ inafunga ncha vizuri |  |  |  |  |  |  |  |  |  |  |  |  |  |  |  |
| 1. Drooping of ear/ kuinama kwa eshisokoro |  |  |  |  |  |  |  |  |  |  |  |  |  |  |  |
| 1. Cob rot resistance/ kustahimili eshisokoro kuoza |  |  |  |  |  |  |  |  |  |  |  |  |  |  |  |
| 1. Resistance to Lodging/Kustahimili kuanguka kwa mmea |  |  |  |  |  |  |  |  |  |  |  |  |  |  |  |
| 1. Foliar disease resistant /Kustahimili magonjwa ya majani |  |  |  |  |  |  |  |  |  |  |  |  |  |  |  |
| 1. Drought resistance/ kustahimili ukame |  |  |  |  |  |  |  |  |  |  |  |  |  |  |  |
| 1. Early maturing/Inakomaa haraka? |  |  |  |  |  |  |  |  |  |  |  |  |  |  |  |
| 1. Yield/Mazao |  |  |  |  |  |  |  |  |  |  |  |  |  |  |  |
| 1. **Overall evaluation** (note: not an average)/kwa ujumla |  |  |  |  |  |  |  |  |  |  |  |  |  |  |  |

*Criteria Importance* ***(0-Not important, 1-Low importance, 2-Medium importance, 3-High importance)***

**A** = like very much/Napenda sana, **B**= like/Napenda, **C**= neither like nor dislike/niko hapo katikati tu, **D**= dislike/Sipendi, **E**= dislike very much/Sipendi kabisa

| **Criteria/ Mizani** |  | **Plot 13** | **Plot 14** | **Plot 15** | **Plot 16** | **Plot 17** | **Plot 18** |  | **Plot 19** | **Plot 20** | **Plot 21** | **Plot 22** | **Plot 23** | **Plot 24** |
| --- | --- | --- | --- | --- | --- | --- | --- | --- | --- | --- | --- | --- | --- | --- |
| 1. Germination/Crop stand |  |  |  |  |  |  |  |  |  |  |  |  |  |  |
| 1. Height/urefu wa mmea |  |  |  |  |  |  |  |  |  |  |  |  |  |  |
| 1. Stalk thickness/ unono wa mti |  |  |  |  |  |  |  |  |  |  |  |  |  |  |
| 1. Resistance to stalk borer/ Kustahimili **stalk borer** |  |  |  |  |  |  |  |  |  |  |  |  |  |  |
| 1. Biomass (for fodder)/ wingi wa majani ya lishe |  |  |  |  |  |  |  |  |  |  |  |  |  |  |
| 1. Barrenness level/ kiwango cha utasa wa mmea |  |  |  |  |  |  |  |  |  |  |  |  |  |  |
| 1. Number of cobs per plant/numbari ya **misakwa** kwa mmea mmoja |  |  |  |  |  |  |  |  |  |  |  |  |  |  |
| 1. Cob size/ ukubwa wa **misakwa** |  |  |  |  |  |  |  |  |  |  |  |  |  |  |
| 1. Good cover of the husk/ inafunga ncha vizuri |  |  |  |  |  |  |  |  |  |  |  |  |  |  |
| 1. Drooping of ear/ kuinama kwa eshisokoro |  |  |  |  |  |  |  |  |  |  |  |  |  |  |
| 1. Cob rot resistance/ kustahimili eshisokoro kuoza |  |  |  |  |  |  |  |  |  |  |  |  |  |  |
| 1. Resistance to Lodging/Kustahimili kuanguka kwa mmea |  |  |  |  |  |  |  |  |  |  |  |  |  |  |
| 1. Foliar disease resistant /Kustahimili magonjwa ya majani |  |  |  |  |  |  |  |  |  |  |  |  |  |  |
| 1. Drought resistance/ kustahimili ukame |  |  |  |  |  |  |  |  |  |  |  |  |  |  |
| 1. Early maturing/Inakomaa haraka? |  |  |  |  |  |  |  |  |  |  |  |  |  |  |
| 1. Yield/Mazao |  |  |  |  |  |  |  |  |  |  |  |  |  |  |
| 1. **Overall evaluation** (note: not an average)/kwa ujumla |  |  |  |  |  |  |  |  |  |  |  |  |  |  |

**Please rank the best 3 plots/ploti 3 bora zaidi: *Plot 1 – 16***

| **Viwango** | **Nambari ya Ploti** | **Sababu ya kwanza ya chaguo lako** | **Sababu ya pili ya chaguo lako** |
| --- | --- | --- | --- |
| Bora zaidi |  |  |  |
| Kadri |  |  |  |
| Ya tatu |  |  |  |

Supplementary Table S1. Pairwise comparison of early-to-intermediate maturity hybrids with two commercial checks

|  | Difference with DUMA43 | | | |  | Difference with PAN4M-19 | | | |
| --- | --- | --- | --- | --- | --- | --- | --- | --- | --- |
| Hybrid | Estimate | St. error | *t* | *p* |  | Estimate | St. Error | *t* | *p* |
| CKH143975 | 1.44 | 0.27 | 5.41 | 0 |  | 1.61 | 0.27 | 6.06 | 0 |
| WE4120 | 1.08 | 0.27 | 4.05 | 0 |  | 1.25 | 0.27 | 4.7 | 0 |
| WE3102 | 1 | 0.27 | 3.77 | 0 |  | 1.17 | 0.27 | 4.41 | 0 |
| WE3101 (CC) | 0.89 | 0.27 | 3.34 | 0 |  | 1.06 | 0.27 | 3.98 | 0 |
| WE3106 | 0.86 | 0.27 | 3.25 | 0 |  | 1.03 | 0.27 | 3.89 | 0 |
| CKH122114 | 0.84 | 0.27 | 3.12 | 0 |  | 1.01 | 0.27 | 3.75 | 0 |
| DLSH103 (CC) | 0.72 | 0.27 | -2.68 | 0 |  | 0.89 | 0.27 | -3.31 | 0 |
| WE4109 | 0.58 | 0.27 | 2.18 | 0.03 |  | 0.75 | 0.27 | 2.82 | 0.01 |
| EMH1101 | 0.47 | 0.27 | 1.76 | 0.08 |  | 0.64 | 0.27 | 2.4 | 0.02 |
| Farmers' check (FC) | 0.15 | 0.27 | 0.57 | 0.57 |  | 0.32 | 0.27 | 1.21 | 0.23 |
| PAN4M-19 (CC) | -0.17 | 0.27 | -0.63 | 0.53 |  |  |  |  |  |

Supplementary Table S2. Pairwise comparison of intermediate-to-late maturity hybrids with two commercial checks

|  | Difference with WH505 | | | |  | Difference with WH509 | | | |
| --- | --- | --- | --- | --- | --- | --- | --- | --- | --- |
| Hybrid | Estimate | St. Error | *t* | *p* |  | Estimate | St. Error | *t* | *p* |
| CKH143770 | 0.65 | 0.34 | 1.91 | 0.06 |  | 0.68 | 0.33 | 2.05 | 0.04 |
| CKH10769 (IC) | 0.18 | 0.33 | 0.53 | 0.6 |  | 0.21 | 0.33 | 0.64 | 0.52 |
| WE3105 | 0.18 | 0.33 | -0.54 | 0.59 |  | 0.21 | 0.33 | -0.65 | 0.51 |
| KM1201 | 0.11 | 0.34 | 0.33 | 0.74 |  | 0.14 | 0.33 | 0.43 | 0.67 |
| WE2106 | 0.1 | 0.33 | 0.29 | 0.77 |  | 0.13 | 0.33 | 0.4 | 0.69 |
| CZH0837 (IC) | 0.07 | 0.33 | 0.2 | 0.84 |  | 0.1 | 0.33 | 0.3 | 0.76 |
| WE1101 (IC) | 0.01 | 0.33 | 0.03 | 0.98 |  | 0.04 | 0.33 | 0.13 | 0.9 |
| WH509 | -0.03 | 0.33 | -0.1 | 0.92 |  |  |  |  |  |
| PHB30G19 (CC) | -0.15 | 0.33 | -0.44 | 0.66 |  | -0.12 | 0.33 | -0.35 | 0.72 |
| WE3104 | -0.16 | 0.33 | 0.47 | 0.64 |  | -0.13 | 0.33 | 0.39 | 0.69 |
| CKH13605 | -0.2 | 0.33 | -0.6 | 0.55 |  | -0.17 | 0.33 | -0.52 | 0.61 |
| Farmers' check | -0.64 | 0.33 | -1.91 | 0.06 |  | -0.61 | 0.33 | -1.86 | 0.06 |


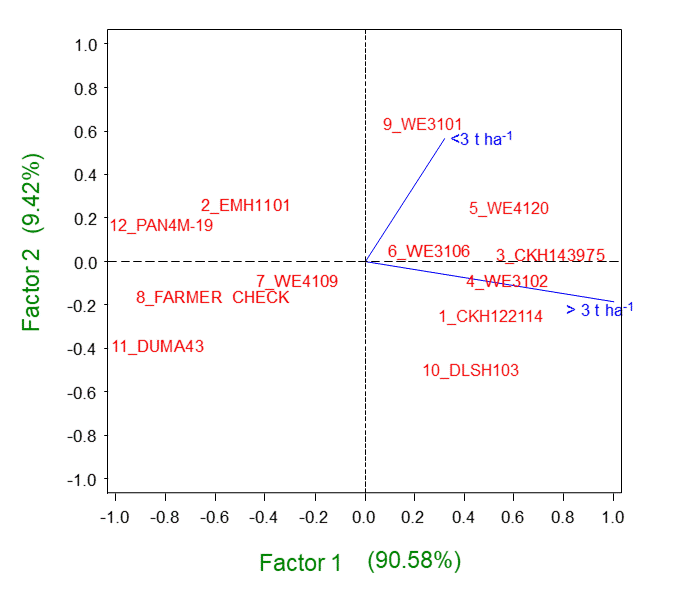


**Supplementary Fig. S1.** Plot of the first (Factor 1) and second (Factor 2) principal components from the site regression model based on grain yield of 12 early-to-intermediate maturity hybrids (marked in red) evaluated under farmer management conditions at 42 environments in East Africa in 2016 and 2017. The two blue lines represent the directions of the PC1 and PC2 scores of the environments representing environments with less (<) than 3 t ha^-1^ and environments with more (>) than 3 t ha^-1^.


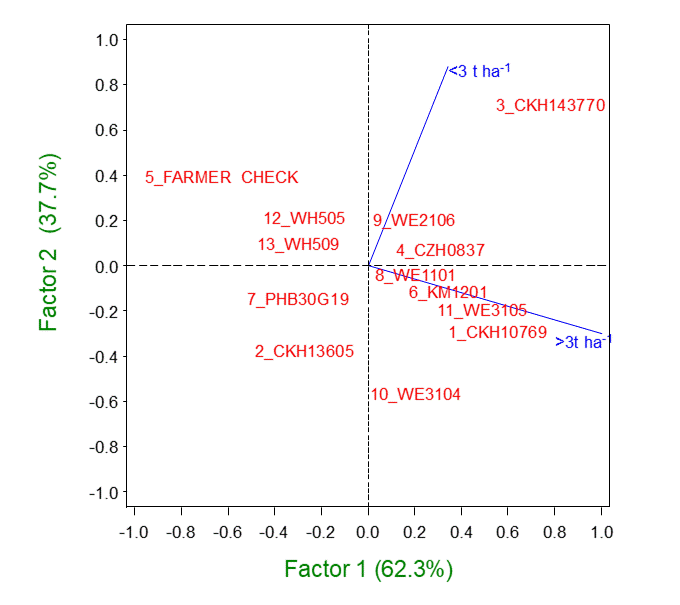


**Supplementary Fig. S2.** Plot of the first (Factor 1) and second (Factor 2) principal components from the site regression model based on grain yield of 12 intermediate-to-late maturity hybrids (marked in red) evaluated under farmer management conditions at 40 environments in East Africa in 2016 and 2017. The two blue lines represent the directions of the PC1 and PC2 scores of the environments representing environments with less (<) than 3 t ha^-1^ and environments with more (>) than 3 t ha^-1^. The cosine of the angle between two hybrid (or management) vectors approximates the correlation between the hybrids (or management) with respect to their interaction. Acute angles indicate positive correlation, with parallel vectors (in exactly the same directions) representing high positive correlation. Obtuse angles (> 90°) represent negative correlations, with opposite directions indicating a negative correlation. Perpendicularity of directions indicates no correlations. Management vectors that point in the same direction as the hybrid vectors have positive interactions (that is, these management factors favored these hybrids), whereas vectors in the opposite direction have negative interactions.
